# Microglial Activation Captured by Diffusion‐Weighted MRI and sTREM2 in Alzheimer’s Disease

**DOI:** 10.1002/alz.090031

**Published:** 2025-01-09

**Authors:** Gohar Zavradyan, Ilana J Bennett, Sneha Padmanaban, Seerat Kang, Sindhuja Kuncharapu

**Affiliations:** ^1^ University of California, Riverside, Riverside, CA USA

## Abstract

**Background:**

Alzheimer’s Disease (AD) is characterized by the accumulation of beta‐amyloid plaques and tau protein tangles and neurodegeneration, with growing interest in the role of neuroinflammation. The neuroinflammatory response to an insult is modulated by microglia, which transition from a resting state marked by ramified, branching processes to an activated stated in which they proliferate, migrate, and swell (processes shorten, somas enlarge). Animal studies have shown that diffusion‐weighted magnetic resonance imaging (MRI) is sensitive to these morphological differences in microglia, with higher diffusion in brain regions experiencing inflammation.

**Method:**

The current study examined the sensitivity of diffusion‐weighted MRI to neuroinflammation in older adults diagnosed with AD, and its relation to an established CSF‐based biomarker of microglial activation (soluble Triggering Receptor Expressed on Myeloid cells 2, sTREM2), using data from the Alzheimer’s Disease Neuroimaging Initiative.

**Result:**

Results reveal significantly higher diffusion (mean diffusivity, MD) in the hippocampus and basal ganglia and higher sTREM2 levels in the AD group relative to the older adults diagnosed with mild cognitive impairment or with no cognitive deficits. In addition, higher MD in the basal ganglia (caudate), but not the hippocampus, was significantly related to sTREM2 levels in the AD group.

**Conclusion:**

These findings are consistent with microglia modulating a neuroinflammatory response in AD and indicate that diffusion‐weighted MRI is sensitive to the swollen morphology of activated microglia.

